# Stress, Workplace Violence, and Burnout in Nurses Working in King Abdullah Medical City During Al-Hajj Season

**DOI:** 10.1097/jnr.0000000000000291

**Published:** 2019-05-20

**Authors:** Ahmad RAYAN, Mo’men SISAN, Omar BAKER

**Affiliations:** 1 PhD, Assistant Professor, Psychiatric and Mental Health Nursing, Zarqa University, Jordan;; 2 BSN, RN, Oncology Specialist Nurse, King Abdullah Medical City, Mecca, Saudi Arabia;; 3 PhD, RN, Associate Professor, College of Nursing, King Saud University, Riyadh, Saudi Arabia.

**Keywords:** stress, workplace violence, burnout, Hajj

## Abstract

**Background:**

The Hajj pilgrimage to Mecca, one of the largest mass gatherings in the world, is associated with various challenges for nurses. One of these challenges is increased levels of workplace violence. Therefore, handling and mitigating workplace violence against nurses during Hajj, when nurses face a higher risk of violence and most experience stress and burnout, is of particular importance.

**Purpose:**

The aims of this study were to identify the types and sources of workplace violence, examine the relationship between burnout in nurses and the variables of stress and workplace violence, and identify from the perspective of nurses measures to effectively handle and mitigate these issues during Hajj season.

**Methods:**

This study used a descriptive correlational design. A convenience sample of 118 nurses completed the Perceived Stress Scale, the Maslach Burnout Inventory, and the modified version of the Joint Programme on Workplace Violence in the Health Sector published by the International Labour Office in Geneva. Data analysis was done using an independent samples *t* test and Pearson product–moment correlation.

**Results:**

One hundred eighteen nurses completed the study. Over two thirds (65%) were female, and 56% reported experiencing at least one type of violence, of which bullying/mobbing, racial harassment, threats, and physical violence accounted for 61%, 15%, 12%, and 12%, respectively. Nurse managers displayed violent behaviors against 54% of the participants. Participants reported high levels of stress and burnout. A positive relationship was found between stress and emotional exhaustion (*r* = .387, *p* < .01). Providing effective security measures and staff training regarding how to deal with violence at the workplace were the main measures identified to help reduce workplace violence.

**Conclusions:**

Providing effective security measures and tailored intervention programs addressing how to deal with violence in the workplace may enable nurses to handle violent behaviors more effectively.

## Introduction

Hajj is one of the main pillars of Islam. Pilgrimage to the holy place of Mecca in Saudi Arabia takes place once a year during the month of “Thulhijah,” which is the last month in the “Hijri” calendar. In 2016, 1.8 million Muslims from all over the world participated in the Hajj (Al Arabiya, 2016). This massive gathering significantly increases the risks for various accident and emergency situations involving the pilgrims. One of the main injuries during Hajj is trauma, which results from factors including walking long distances and motor vehicle accidents in congested areas full of pilgrims and vehicles (Ahmed, Arabi, & Memish, 2006). In addition, the immensely crowded settings increase the risk of transmitting infections such as meningococcal disease and pneumonia (Alsafadi, Goodwin, & Syed, 2011). Dealing with these conditions adds extra burdens on and increases the stress levels of nurses who provide care to Hajj pilgrims.

According to the latest study conducted during the Hajj period, most nurses were found to have limited knowledge and awareness regarding disaster preparedness plans and strategies for managing mass gathering disasters (Alzahrani & Kyratsis, 2017). Subsequently, there is a need to identify nurses’ concerns and experiences associated with mass gathering during the Hajj such as workplace violence, stress, and burnout (Alsaqri, 2014; Tsouros & Efstathiou, 2007). In fact, addressing nurses’ concerns during Hajj is of special importance and has international significance, as nurses working during the Hajj are of different ethnic and national backgrounds. The current study not only investigates workplace violence, stress, and burnout but also identifies from the perspective of nurses the measures that may help effectively handle and mitigate workplace violence during Hajj season. The findings may be helpful in the development of special intervention programs to control workplace violence in the future.

King Abdullah Medical City in the Holy Capital of Mekkah (KAMC-HC) is one of the largest medical cities in Saudi Arabia that provides medical care to pilgrims during Hajj season. KAMC-HC has 350 beds covering all specialties and employs nurses of different nationalities. Healthcare manpower at KAMC-HC is not increased during Hajj season. Subsequently, to meet the increased work demands during this period, nurses at KAMC-HC usually work 12 hours a day for at least 15 days or more consecutively. Long shifts and stressful work conditions understandably increase the stress and burnout levels of nurses (Dall’Ora, Griffiths, Ball, Simon, & Aiken, 2015; S. Wu, Zhu, Wang, Wang, & Lan, 2007). Furthermore, during Hajj season, many nurses are reassigned from their normal departments to emergency rooms (ERs), operating rooms, or intensive care units (ICUs). Most standby nurses are not familiar with the daily routine of these critical care units, which may further aggravate their stress levels.

Violence in the workplace is another factor that contributes to stress in nurses and decreases their work productivity (Gates, Gillespie, & Succop, 2011). Violence in the workplace against nurses is a worldwide phenomenon that is largely independent of ethnic/national background (Spector, Zhou, & Che, 2014). However, incidents of violence against nurses typically increase during Hajj because of shortages in nursing staff, language barriers (inability to understand the language of patients who come from all over the world), and shortages of security personnel (Alkorashy & Al Moalad, 2016; Mohamed, 2002). Because of their close contact with patients and their families, nurses face a higher risk of workplace violence than other healthcare professionals (Kwok et al., 2006). It is well documented in the literature that violence in the workplace contributes to work dissatisfaction, decreased productivity, high rates of stress and turnover, and burnout (Gates et al., 2011; Laschinger & Grau, 2012; Stimpfel & Aiken, 2013). To date, limited statistics are available regarding the prevalence of workplace violence against nurses during Hajj season.

A recent study conducted in Saudi Arabia reported that almost 50% of nurses experienced violence at the workplace in 2015 (Alkorashy & Al Moalad, 2016). Alkorashy and Al Moalad (2016) recommended developing specific policies for preventing workplace violence. However, additional preventive measures related to security measures in the workplace and preparing staff to deal effectively with the potential violent incidences during Hajj may be necessary. However, little is known regarding how workplace violence relates to levels of stress and burnout in nurses working during Hajj season, making it difficult to tailor and conduct intervention programs to address this issue and improve working conditions. Therefore, the purpose of this study was to examine the relationship between workplace violence, stress, and burnout in nurses working at KAMC-HC. The related objectives were

- to identify the types and sources of workplace violence toward nurses working at KAMC-HC during Hajj season;
- to identify from the perspective of nurses measures to effectively handle and mitigate workplace violence at KAMC-HC;
- to assess levels of stress and burnout among nurses working at KAMC-HC during Hajj season; and
- to assess the relationships among workplace violence, stress, and burnout in nurses.

## Methods

Data collection was completed using a self-administered questionnaire during the rituals of Al-Hajj from September 1 to 15, 2016. For the purpose of this study, a descriptive, cross-sectional, and correlational design was used. Institutional review board approval was obtained from the KAMC (Approval number 2016/58). Confidentiality was guaranteed for all study participants. Anonymity and autonomy were assured for all participants, and all were free to participate or withdraw from the study at any time without consequence. In addition, participants were briefed about the purpose of the study before data collection. Data were collected by the first author.

### Participants

All of the participants in this study were working at KAMC-HC. According to the latest statistics published by the Saudi Arabia Ministry of Health in 2016, the total number of nurses (including midwives) working in Saudi Arabia includes about 180,821 of various nationalities. More than half of these (101,256) work for the Ministry of Health, representing about 57.6% of the nursing workforce. The approximately 900 nurses working at KAMC-HC are nationals of 10 countries, including the Philippines, Jordan, India, Egypt, the United States, Yemen, Lebanon, Malaysia, Pakistan, and Turkistan. The largest percentage (38.3%) are from the Philippines.

On the basis of a G*Power calculation for an independent samples *t* test with an alpha level of .05, a power of 0.80, and a medium effect size, 102 participants were needed. To obtain more power, the sample size was increased to 120. Inclusion criteria were as follows: being a full-time registered nurse working at KAMC-HC during Al-Hajj season, having over 1 year of experience at KAMC-HC, and working at patient bedsides during Al-Hajj season. Data were not collected from nurses who did not meet the inclusion criteria, including those having less than a year of experience at KAMC-HC, not providing direct care to patients, or not working during Al-Hajj season. Five hundred ninety of KAMC-HC’s 900 nurses met the inclusion criteria. A convenience sample of 120 registered nurses working at KAMC-HC was recruited. About 11.2% (66) had been reassigned to work in other departments such as ICU or ER during Hajj season.

### Instruments

A demographic questionnaire and three scales, including the modified version of the Joint Programme on Workplace Violence in the Health Sector published by the International Labour Office, the Perceived Stress Scale (PSS), and Maslach Burnout Inventory (MBI), were included in the questionnaire. A pilot study was conducted on 31 nurses to assess questionnaire validity and reliability. The questionnaire took about 10–15 minutes to complete during the pilot study, and all of the subscales earned a Cronbach’s alpha of at least .72.

### Demographic Questionnaire

The demographic questionnaire gathered data on age, gender, education level, years of experiences, and marital status.

### Questionnaire on the Prevalence, Types, Sources, and Measures Used to Handle Workplace Violence

A survey developed by the “Joint Programme on Workplace Violence in the Health Sector” of the International Labour Office, International Council of Nurses, World Health Organization, and Public Services International (2003) was used in this study. This survey investigates both the types of workplace violence, including psychological violence (threats, bullying/mobbing, and racial harassment) and physical violence, and the sources of violence, including managers, colleagues, and other sources (e.g., visitors, police). Examples of measures taken to handle workplace violence include security measures and restricting public access. In addition, participants had the option to select more than one alternative. Furthermore, participants with no experience of workplace violence were given the chance to select answers concerning the most effective measures to reduce workplace violence based on incidences of violence that they had witnessed. This survey measure has excellent psychometric properties and has been used in countries such as Lebanon, South Africa, Brazil, Portugal, Australia, and Thailand (Di Martino, 2002). In the current study, Cronbach’s alpha coefficients for the types, sources, and measures for handling workplace violence were adequate to high, ranging from .76 to .93.

### The Perceived Stress Scale

In this study, the 10-item PSS (PSS-10) was used to measure the level of stress of the participants. The PSS is considered one of the most commonly used psychological tools to measure stress perception in individuals (Cohen & Williamson, 1988). It comprises multiple-choice questions that are scored on a 5-point Likert scale (0 = *never* to 4 = *very often*). Items 4, 5, 7, and 8 are reverse scored. The PSS-10 is a reliable scale, and the original total scale has a reported Cronbach’s alpha of .78 (Cohen & Williamson, 1988). Recently, the measure has shown evidence of excellent psychometric properties in various populations (Andreou et al., 2011; Chaaya, Osman, Naassan, & Mahfoud, 2010; Reis, Hino, & Añez, 2010). Cronbach’s alpha coefficients for the PSS-10 range from .78 to .91 (Cohen, Kamarck, & Mermelstein, 1983; Cohen & Williamson, 1988; Ezzati et al., 2014; S. M. Wu & Amtmann, 2013). Total scores range from 0 to 40, with 0–7 indicating very low stress, 8–11 indicating low stress, 12–15 indicating average stress, 16–20 indicating high stress, and 21 or over indicating very high stress (Cohen et al., 1983).

### Maslach Burnout Inventory

The MBI was used to measure the level of burnout perceived by the participants. The MBI, a 22-item instrument developed by Maslach and Jackson (1981), comprises three subscales. The Emotional Exhaustion (EE) subscale has nine items that address the sense of emotional exhaustion at work, the Personal Accomplishment (PA) subscale has eight items that address the sense of reduced professional competence and decreased positive individual interactions at work, and the Depersonalization (DP) subscale has five items that address the sense of decreased personal involvement with people and emotional disinterest in one’s job. The MBI includes frequencies ranging from 0 (never) to 6 (everyday), with low PA scores and high DP and EE scores indicating job burnout. Total EE subscale scores below 17, between 17 and 26, and higher than 26 represent low, moderate, and high levels of job burnout, respectively. Total DP subscale scores below 7, between 7 and 12, and higher than 12 represent low, intermediate, and high levels of burnout, respectively (Maslach, Schaufeli, & Leiter, 2001). Finally, total PA subscale scores below 32, between 32 and 38, and higher than 38 represent high, intermediate, and low levels of burnout, respectively. Maslach and Jackson (1981) reported evidence supporting that the 22-item MBI has both high reliability and validity. The reliability coefficients for the subscales were .89 for EE, .74 for PA, and .77 for DP (Maslach & Jackson, 1981). Furthermore, Iwanicki and Schwab (1981) reported Cronbach’s alpha values of .90, .76, and .76 for the EE, DP, and PA subscales, respectively. The scores for each subscale of the MBI should be considered separately and are not suitable for combining into a single total score.

### Data Analysis

Data were analyzed using SPSS Version 21 (IBM, Armonk, NY, USA). Descriptive statistics were used to present the sample characteristics, and the independent samples *t* test and Pearson product–moment correlation were used to examine the relationships among workplace violence, stress, and nurse burnout.

## Results

### Demographic Data

Of the 120 nurses who were invited, 118 nurses completed and returned the study questionnaires, representing a response rate of 98%. The mean age of the participants was 29.14 years. About 65% were female, and most (71%) were married. About 43% worked in close units, and most (91.5%) held a bachelor degree in nursing as their highest level of academic achievement. Mean experience was 7.53 (*SD* = 6.8) years. Thirty-seven (31%) had less than 5 years of experience, 64 (55%) had 5–10 years of experience, and 17 (14%) had over 10 years of experience. The largest numbers of participants were from the Philippines (37%), India (28%), and Pakistan (13%), with fewer numbers from other countries, including Jordan, Egypt, Yemen, Lebanon, and Turkistan (22%).

### Types and Sources of Workplace Violence Toward Nurses

About 56% of the participants reported experiencing workplace violence during the last 6 months. In terms of types of workplace violence, about 61%, 15%, 12%, and 12% of the participants were exposed to bullying/mobbing, racial harassment, threat, and physical violence, respectively. About 54% of the participants reported experiencing violence from their managers, whereas 32% and 14% reported experiencing violence from colleagues (other nurses) and other sources (e.g., visitors, police), respectively.

### Measures Deemed by Nurses as Helpful to Handling Workplace Violence

Table 1 presents the measures recommended by nurses as helpful in handling workplace violence. The most commonly reported included providing effective security measures in the workplace and providing staff training. The least commonly reported measure was restricting public access to workplaces.

**TABLE 1.**
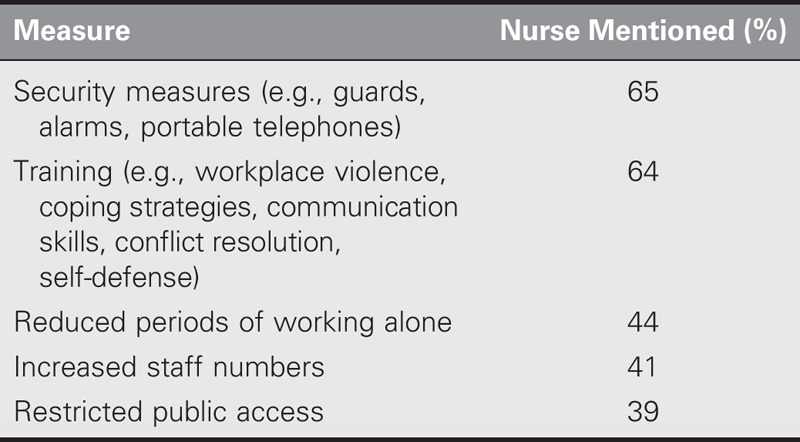
Helpful Measures for Handling Workplace Violence From the Perspective of Nurses

### Levels of Burnout and Stress

Table 2 presents the levels of stress and burnout, respectively, for each subscale of the MBI. The participants reported high levels of psychological stress, emotional exhaustion, personal accomplishment, and depersonalization, reflecting high overall levels of burnout.

**TABLE 2.**
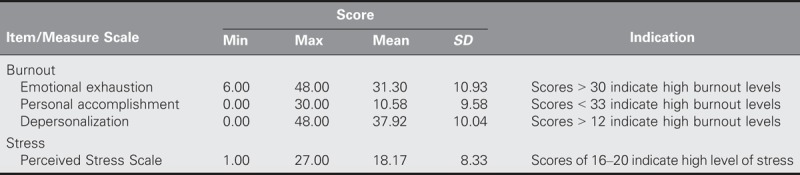
Levels of Burnout and Stress Among Nurses

### Relationships Among Workplace Violence, Stress, and Burnout

An independent samples *t* test was used to examine if there was a significant difference in stress and burnout based on experiencing violence in the workplace. Expectedly, nurses who were exposed to violence at their place of work reported higher levels of stress and burnout than those who did not. However, stress levels were significantly different only between nurses who were exposed to violence and those who were not (Table 3). Pearson product–moment correlation indicated a significantly positive relationship between stress and the EE subscale of MBI (*r* = .387, *p* < .01). However, stress was not significantly correlated with either the PA or DP subscale of MBI (*r* = .022, *p* > .05; *r* = .015, *p* > .05, respectively).

**TABLE 3.**
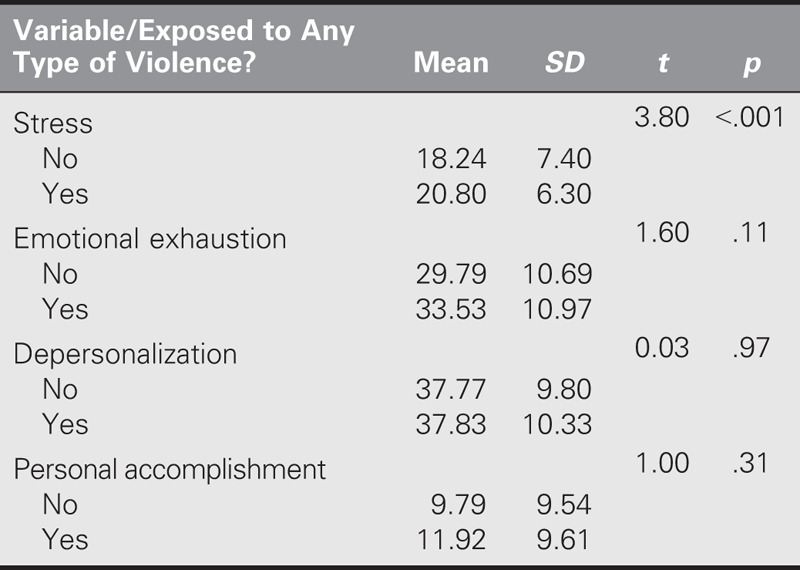
Differences in Stress and Burnout Based on Having Experienced Violence in the Workplace (*N* = 118)

## Discussion

The current study investigated the relationships among workplace violence, stress, and burnout in nurses at KAMC-HC during Al-Hajj season 1437 AH. Prevalence of workplace violence, types of violence, and the measures to reduce violence were highlighted. To the best of authors’ knowledge, this is the first study to explore the relationships among workplace violence, stress, and nurse burnout during Hajj season. The response rate of 98% is considered favorable given the exploratory nature of this topic. The results indicated that participants who were subject to violence in the workplace experienced higher levels of stress than participants who were not. In addition, a positive relationship was found between stress and the EE subscale of MBI.

Overall, the participants reported very high levels of psychological stress and burnout. In addition, more than half reported having experienced workplace violence in the most recent month. As nursing is a high-pressure profession, high stress levels is an expected outcome, especially during heavy workload periods such as Hajj season. In fact, stress-free environments are rare in the nursing profession. However, some factors such as high workloads and poor working conditions make nurses vulnerable to physical and psychological stress (Geiger-Brown et al., 2012; Trinkoff et al., 2011). Nurse managers should monitor stress levels among their employees, identify stress-contributing factors, and adopt measures to alleviate stress such as providing appropriate work schedules for nurses and improving communications between them (Carr, Kelley, Keaton, & Albrecht, 2011).

In this study, the participants who had been exposed to workplace violence of any type during Hajj season tended to be more stressed than those who had not. Most of the participants were not Arabic speakers, which may affect their levels of stress and burnout, and workplace violence experience. During Hajj season, most nurses are required to work additional hours to compensate for staff shortages while dealing with higher frequencies of emergencies and accidents. According to the literature, mandatory overtime may negatively affect the psychological well-being of nurses because of lack of control (Lobo, Fisher, Ploeg, Peachey, & Akhtar-Danesh, 2013).

Another important finding of this study was that 54% of the participants reported experiencing workplace violence from their managers, which is relatively high when taking into consideration that managers should be a source of support and strength for their nursing staff. In addition, psychological violence was more prevalent than physical violence. Nurse managers should not ignore the huge impact of stress on the functioning of an organization. In addition, these managers should be aware of the relationship between experiencing violence in the workplace and stress among nurses. In fact, both psychological and physical violence in the workplace seriously influence the health and well-being of nurses (Gates et al., 2011).

The participants in this study reported higher levels of burnout compared with a study of multinational nurses working in Saudi Arabia (Al-Turki et al., 2010). Worldwide, comparative studies have shown burnout to be a significant problem inside the world of nursing, with nurses having the highest risk of burnout of any category of healthcare provider (Bernardi, Catania, & Marceca, 2005). The high levels of burnout found in this study may be associated with high levels of stress during Hajj season. In a sample of 1,363 nurses working in hospitals, workload correlated significantly with emotional exhaustion (Greenglass, Burke, & Fiksenbaum, 2001).

Previous research highlighted the importance of developing specific policies for preventing workplace violence (Alkorashy& Al Moalad, 2016). However, providing effective security measures and staff training on how to deal with violence at the workplace were considered by the participants in this study to be the most helpful actions for reducing workplace violence. Thus, these strategies should be given priority consideration when dealing with workplace violence during periods of mass gatherings such as Hajj.

### Strengths and Limitations

This study highlighted the contemporary and serious issues faced by nurses of different nationalities working during Hajj season. Because the participants were recruited from one large hospital during a specific period, it may be difficult to generalize the outcomes beyond the limitations of place and situation. However, this study reflects a phenomenon of concern in nursing, as nurses worldwide experience higher workloads during crisis situations. Furthermore, this study explored the relationships among workplace violence, stress, and nurse burnout, which has important implications for nursing practice and research.

### Conclusion and Recommendations

This study offers important recommendations for the nursing profession. Preventing workplace violence is more important than providing interventions. In addressing the causes of the problem, it will be necessary to effectively address the related causal factors. Nurses require training to handle violent behaviors effectively. Managers have the primary responsibility to provide adequate staffing and safety measures and to assign sufficient security personnel to critical care areas such as the ER and ICU. Moreover, each hospital should maintain policies addressing violence in the workplace. The policy concerning violence should be appraised on an annual basis (Rayan, Qurneh, Elayyan, & Baker, 2016).

Further research is necessary to further investigate the effectiveness of applying specific strategies to reduce stress levels and burnout and to decrease the incidence of workplace violence. Information concerning circumstances that initiate violent events, burnout, and possible stressors may highlight the results. In addition, it is essential to elicit the predictors and indicators of rising levels of stress or burnout in nurses.
